# A practical and economical method for frontal sinus reconstruction after frontal craniotomy: A single-center experience with 140 patients

**DOI:** 10.3389/fsurg.2022.919276

**Published:** 2022-07-20

**Authors:** Youwei Guo, Xianyong Fu, Wen Yin, Zhipeng Jiang, Yirui Kuang, Zhaoping Wu, Yudong Cao, Jun Tan, Xing-jun Jiang

**Affiliations:** ^1^ Department of Neurosurgery, Xiangya Hospital, Central South University, Changsha, China; ^2^ Department of Neurosurgery, Hospital of the Chinese People’s Liberation Army, Third Military Medical University, Chongqing, China; ^3^ National Clinical Research Center for Geriatric Disorders, Xiangya Hospital, Central South University, Changsha, China

**Keywords:** gelfoam, vascularized pericranial flap, frontal sinus, reconstruction, cerebrospinal fluid leak

## Abstract

**Background:**

Frontal sinus exposure is a common consequence of frontal craniotomy. Cerebrospinal fluid leakage and infection are the major postoperative complications that may occur as a result of the open frontal sinus. The successful filling of the open frontal sinus provides an approach to prevent significant complications caused by frontal sinus exposure.

**Objective:**

This article describes a new technique to reconstruct the exposed frontal sinus cavity with the combined application of gelatin sponge and a vascularized pericranial flap.

**Methods:**

A total of 140 patients underwent frontal sinus reconstruction using gelfoam and vascularized pericranial flaps from 2016 to 2021. Gelatin sponge was used to fill the frontal sinus, and a vascularized pericranial flap was used to cover the frontal sinus when the bone flap was retracted.

**Results:**

Postoperative cerebrospinal fluid leakage and infection did not occur in any patient.

**Conclusion:**

Our results validated the effectiveness of our technique in the prevention of exposed frontal sinus-related postoperative complications.

## Introduction

The subfrontal approach is an effective treatment for anterior skull base tumors, sellar region lesions, and anterior cerebral artery aneurysms (1–3). The frontal sinus is frequently opened during a craniotomy when it is gasified. Various postoperative complications may occur due to the opening of the frontal sinus, including cerebrospinal fluid (CSF) leakage and intracranial infection (4–7). To avoid such complications, it is necessary to completely block the connection between the frontal sinus and intracranial contens.

Neurosurgeons have tried various methods to completely block the connection by using autologous tissue (e.g., fat, fascia, muscle, or pericranial flap) or synthetic material (e.g., polymethyl methacrylate hydroxyapatite cement and bone wax) (1, 8–16). Autologous tissue is readily available, and there is no rejection response. However, the acquisition of autologous tissue may result in new damage sites. The most implantable materials cannot be absorbed and are relatively expensive. Therefore, an effective and economical method for frontal sinus reconstruction is needed in order to effectively block the connection between the frontal sinus and intracalvarium.

In this study, we described a practical and economical approach to reconstruct the frontal sinus, a method that has not yet been reported. A combined strategy of gelatin sponge and the vascularized pericranial flap was used to treat an open frontal sinus during frontal craniotomy. Gelatin sponge was used to fill the frontal sinus, and a vascularized pericranial flap was used to cover the frontal sinus when the bone flap was retracted. The results gathered from 140 patients indicated that our technique is a safe, practical, and economical method to reconstruct the frontal sinus.

## Materials and method

### Patients

This study included 140 patients (71 men and 69 women; age range, 9–70 years; mean age, 45.1 years) who underwent frontal craniotomy with frontal sinus exposure at the anterior skull base, the base of the frontal lobe or sellar lesions, including craniopharyngioma (63 cases), anterior skull base meningioma(37 cases), glioma (11 cases), pituitary adenoma (13 cases), germ-cell tumor (6 cases). Rathke cyst (2 cases) and other (8 cases) (Table 1), from June 2013 to April 2021 at Xiangya Hospital of Central South University. In all 140 patients underwent frontal sinus reconstruction with the use of gelatin sponge and vascularized pericranial flap as described subsequently. We retrospectively analyzed the demographics and postoperative complications of the 140 patients in the study group.

**Table 1:** Distribution of lesion types.

Lesion	No. of case
Craniopharyngioma	63
Meningioma	37
Glioma	11
Germ-cell tumor	6
Pituitary adenoma	13
Rathke cyst	2
Haemangiopercytoma	2
Cavernous hemangioma	2
Metastatic tumor	2
Arachnoid cyst	1
Cholesteatoma	1
	140

### Frontal sinus reconstruction

**Figure 1:**
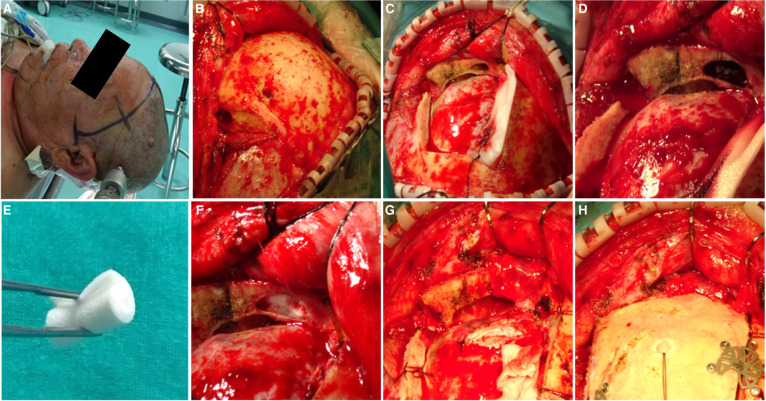
The process of frontal sinus reconstruction. (**A**) A coronal skin incision is made in the hairline. (**B**) One burr hole was made at the Key hole. (**C**) The craniotomy was performed in a square shape. (**D**) The open frontal sinus mucosa was peeled off from the inner wall of the frontal sinus and pushed toward to the frontonasal duct. (**E**) Gelfoam was rolled into a cylindrical shape. (**F**) The opened frontal sinus was completely obliterated with gelfoam. (**G**) The vascularized pericranial flap covered the roof wall of the frontal sinus. (**H**) The vascularized pericranial flap helped to fix the bone flap once it was retracted.

The patient was placed in the supine position. The head was moderately placed backward or forward, depending on the location of the lesion. One gram of ceftriaxone sodium was administered before skin incision and then applied every three hours. The coronal skin incision was made in the hairline (Figure 1A). The skin flap was separated from the periosteum and subgaleal connective tissue. One burr hole was made at the keyhole after separating the temporalis partly from the supratemporal line (Figure 1B). The inferior craniotomy margin was close to the anterior skull base, and the posterior wall of the frontal sinus was exposed in all patients.

In all 140 cases, the mucosal membrane of the frontal sinus was exposed (Figure 1C). disinfected with povidone-iodine. The mucosal membrane of the frontal sinus was then peeled off from the inner wall of the frontal sinus and pushed toward the frontonasal duct (Figure 1D). The hemorrhagic spots on the frontal sinus wall were eliminated by electrocoagulation. The gelatin sponge was folded into cylindrical shape (Figure 1E), and the frontal sinus cavity was filled with the gelatin sponge (Figure 1F), the gelatin sponge should be tightly compressed and make sure there is no residual space. After lesion removal and dura closure, the pericranium and loose areolar tissue were separated from the galea aponeurotica. The size of the vascularized pericranial flap, which was larger than the opened roof wall of the frontal sinus. The vascularized pericranial flap covered the open sites of the frontal sinus (Figure 1G). and helped to fix the bone flap (Figure 1H). The skin was then sutured layer by layer. The process of frontal sinus reconstruction was showed in video (Supplementary Video S1).

### Postoperative management

Antibiotics (ceftriaxone sodium 2 g/day) were administered for two days to prevent infection. Rehabilitation and meals were provided after once the patients regained consciousness, and patients were discharged once they are in good health. Magnetic resonance imaging was performed during follow-up.

## Results

### Surgical outcomes

Total tumor resection was achieved in all paitents. None of the patients died perioperatively. Postoperative fever occurred in 42 patients. Only two paitents had transient intracranial infections that recovered after antibiotic treatment. In the remaining cases, fever may have been associated with hemorrhagic cerebrospinal fluid. All patients with fever were well by the time they were discharged. Postoperative CSF leakage was not observed in any patient.

### Follow up

**Figure 2:**
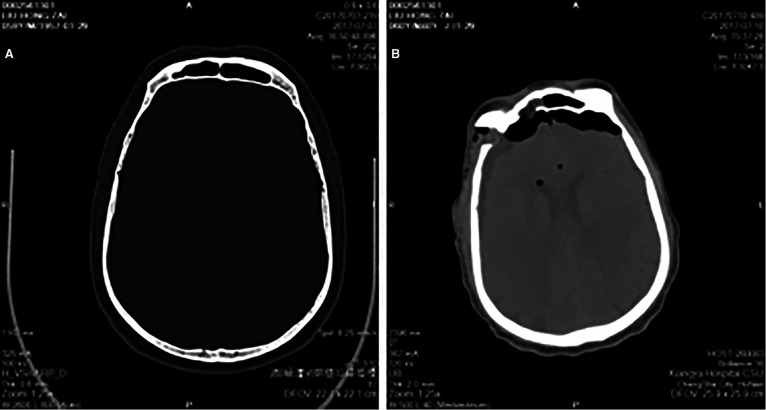
Pre- (**A**) and post-operative (**B**) CT scaning at frontal sinus level.

**Figure 3:**
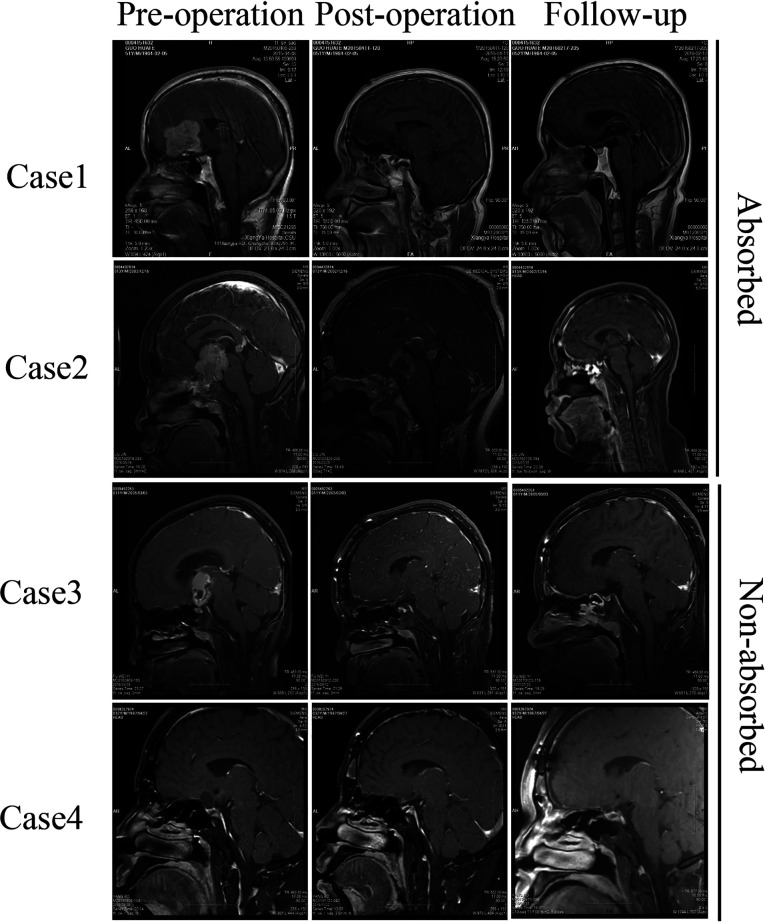
Pre/post- operative and follow-up MRI study. The pre-operative MRI showed the lesion at the anterior skull base and the frontal sinus. The post-operative MRI showed the open frontal sinus filled with gelfoam. The follow-up MRI showed that the gelfoam was absorbed or not absorbed during follow-up.

All patients were subjected to computed tomography scanning instantaneously and magnetic resonance imaging two days after surgery. Computed tomography scanning showed that the exposed frontal sinus was filled by gelatin sponge (Figures 2A,B). The results from magnetic resonance imaging further confirmed this observation (Figure 3). All patients were subjected to 3–80 months (mean, 41 months) postoperative follow-up. More importantly, out of 108 cases of performed magnetic resonance imaging, in 36 cases of the gelatin sponges observed were partially or wholly absorbed. Representative absorbed or unabsorbed cases are shown in Figure 3. This result has not been reported in other studies.

## Discussion

Mismanaged frontal sinus exposure can potentially result in severe and long-lasting sequelae. CSF leakage and frontal sinus infection are the major complications after frontal sinus exposure during frontal craniotomy. These conditions may further advance into meningitis and intracranial abscesses, which leads to poor postoperative outcomes and prolonged hospital stays. There are several techniques for frontal sinus reconstruction; those studies demonstrate no significant difference in the complication rates after the surgical repair of the frontal sinus, especially in those large series (Table 2). Compare with other studies, we describe a more practical, effective, less damaging, and economical technique for frontal sinus reconstruction. In this study, we utilized a combined strategy to reconstruct the exposed frontal sinus, in which the gelatin sponge was used to fill the exposed frontal sinus and a vascularized pericranial flap was used.

**Table 2:** Frontal sinus reconstruction methods with large series cases.

Type	Materials	Author	Year	No. of case	Combine with other material	Complication (CSF, frontal sinusitis, intracranial Infection mucous cyst)
Autologous material	Fat	Rainer Weber et al. (11)	2000	82	Cartilage, Connective tissue and fibrin glue	Mucous cyst (4 cases)
	Fat	Satoru Takeuchi et al. (17)	2015	103	Fibrin glue	Intracranial infection (1 case)
	Fat	Ittichai Sakarunchai et al. (18)	2016	107	Fibrin glue	None
	Bone	Satoyuki Ito et al. (14)	2003	11	Fibrin glue	None
	Pericranial flap	Alexander Donath et al. (19)	2006	19	None	CSF (1 case)
Synthetic material	Gelfoam	Zhou et al. (20)	2013	118	Aural and encephalic glue	None
	Gelfoam	Yasuo Murai et al. (21)	2014	51	Fibrin glue and bone graft	None
	PMPA	Jin Matsuura et al. (12)	2019	52	None	None
	Hydroxyapatite cement	Guy J. Petruzzelli et al. (22)	2001	11	None	None

Autologous tissues, which include fat, muscle, or pericranial flaps, are commonly used in the obliteration of the frontal sinus (15). Although autologous tissues are considered as the ideal material for the frontal sinus reconstruction, those tissues have their own characteristics. Fat has a wide range of sources and strong plastic characteristics, which makes it widely used in the frontal sinus obliteration. However, the use of fat could cause additional damage to the donor sites and prolonge the operative time (11, 17, 18, 23). The pericranial flap is commonly used for anterior skull base reconstruction (24–26). Few studies have reported the application of pericranial flaps for frontal sinus reconstruction. Alexander et al. covered the frontal sinus with a pericranial flap alone in 19 patients, and one patient presented with cerebrospinal fluid leakage during hospitalization (19). Rainer et al. combined fat, fibrin glue, and a pericranial flap to the obliteration of frontal sinus, no patient developed to CSF (11). The application of pericranial flap alone to block the open frontal sinus is not very reliable. In our study, a single piece of the vascularized pericranial flap was sufficient to cover the open frontal sinus, and the damage was minimal when compared with the application of other autologous tissues for the obliteration of the frontal sinus. The vascularized pericranial flap is the second layer for the frontal sinus obliteration. More importantly, the vascularized pericranial flap will prevent the transfer of infection from the frontal sinus to the intracranial cavity after gelatin sponge is absorbed.

Synthetic materials, sunch as polymethyl methacrylate, bone max, and hydroxyapatite cement, have also been attempted in the obliteration of the frontal sinus (12, 22, 27). Polymethyl methacrylate and hydroxyapatite cement are more expensive than traditional techniques, and these materials do not completely seal the open frontal sinus. Bone wax has a long history of application in neurosurgery (28). It cannot be absorbed and may cause chronic inflammation and poor wound healing (27). The absorbable, economic, and porous properties of gelatin sponge make it an ideal material for frontal sinus obliteration (20, 21).

Gelatin sponge is commonly used in neurosurgery as a hemostatic agent. It is a porous, compressible, and pliable material derived from pork skin gelatin, which can absorb fluids (29). In this study, the dry gelatin sponge was compacted and rolled into a cylindrical shape, and the frontal sinus was completely filled with the gelatin sponge. The dry gelatin sponge will expand after absorbing blood. In a previous study, Zhou et al. used medical aural and encephalic glue-soaked gelatin sponge for frontal sinus repair (20). This method also efficiently reconstructs the frontal sinus without any infection-related complications. However, they only used a correctly sized piece of gelatin sponge injected with encephalic glue to seal the open frontal sinus. Encephalic glue solidifies quickly, and it is difficult for gelatin sponges to fit the inner wall of the frontal sinus with water tightness. Compared with this study, our approach tends to be a more practical and low-cost method for the repair of the open frontal sinus. In addition, the vascularized pericranial flap at the roof of the frontal sinus is a reliable way to seal the open frontal sinus once the gelatin sponge is absorbed. Our combined strategy is a promising approach to reduce the long-term infection-related complications caused by frontal sinus sources.

## Conclusion

We describe the combined application of the gelatin sponge and vascularized pericranial flap to treat an open frontal sinus during frontal craniotomy. The case series is the largest in English literature. The combined application of the gelatin sponge and the vascularized pericranial flap is a practical, effective, less damaging, and economical technique. Postoperatively, all patients recovered without any complications. Therefore, our study concluded that gelfoam combined with a vascularized pericranial flap is a practical and economical method for frontal sinus reconstruction.

## Data Availability

The raw data supporting the conclusions of this article will be made available by the authors, without undue reservation.
